# RETRACTED: Wojtaszek et al. Physicochemical, Antioxidant, Organoleptic, and Anti-Diabetic Properties of Innovative Beef Burgers Enriched with Juices of Açaí (*Euterpe oleracea* Mart.) and Sea Buckthorn (*Hippophae rhamnoides* L.) Berries. *Foods* 2024, *13*, 3209

**DOI:** 10.3390/foods15152703

**Published:** 2026-07-31

**Authors:** Anna Wojtaszek, Anna Marietta Salejda, Agnieszka Nawirska-Olszańska, Aleksandra Zambrowicz, Aleksandra Szmaja, Jagoda Ambrozik-Haba

**Affiliations:** Faculty of Biotechnology and Food Sciences, Wrocław University of Environmental and Life Sciences, Chełmońskiego 37 Str., 51-630 Wrocław, Poland; anna.wojtaszek98@interia.pl (A.W.); agnieszka.nawirska-olszanska@upwr.edu.pl (A.N.-O.); aleksandra.zambrowicz@upwr.edu.pl (A.Z.); aleksandra.szmaja@upwr.edu.pl (A.S.); jagoda.ambrozik-haba@upwr.edu.pl (J.A.-H.)

The journal retracts the article titled “Physicochemical, Antioxidant, Organoleptic, and Anti-Diabetic Properties of Innovative Beef Burgers Enriched with Juices of Açaí (*Euterpe oleracea* Mart.) and Sea Buckthorn (*Hippophae rhamnoides* L.) Berries” [1], cited above. 

Following publication, the authors contacted the Editorial Office regarding the accuracy of the Institutional Review Board statement reported in this publication [1].

In accordance with the journal’s standard procedures, an investigation was conducted by the Editorial Office in collaboration with the Editorial Board. The investigation determined that the published statement indicating that ethics approval for the study had been obtained was inaccurate and that no valid ethics approval for this study could be provided retrospectively. The investigation further concluded that the study did not comply with the journal’s policy on Research Involving Human Subjects. Consequently, the Editorial Board decided to retract this paper [1] as per MDPI’s retraction policy. 

This retraction was approved by the Editor-in-Chief of the journal *Foods*.

The authors have been informed of this retraction and have indicated their agreement with this decision.
